# Using the RIGHT statement to evaluate the reporting quality of clinical practice guidelines in traditional Chinese medicine

**DOI:** 10.1371/journal.pone.0207580

**Published:** 2018-11-16

**Authors:** Xia Yun, Chen Yaolong, Zeng Zhao, Zhou Qi, Wang Yangyang, Xie Runshen, Xie Xiuli, Li Hui

**Affiliations:** 1 Guangzhou Chinese Medical University, Guangzhou, Guangdong, China; 2 Guangdong Province Hospital of Chinese Medicine, Guangzhou, Guangdong, China; 3 Lanzhou University, Lanzhou, Gansu, China; Flinders University, AUSTRALIA

## Abstract

**Objective:**

To evaluate the reporting quality of clinical practice guidelines (CPGs) in traditional Chinese medicine (TCM).

**Methods:**

A systematic search was undertaken to extract CPGs for TCM. The RIGHT (Reporting Items for practice Guidelines in Healthcare) statement was used to calculate scores for the reporting quality in terms of domains and items, followed by a subgroup analysis of the results and determination of the correlation between the RIGHT and AGREE II (Appraisal of Guidelines for Research and Evaluation II) scores.

**Results:**

Overall, 539 TCM CPGs were included. (1) The mean scores (Med, IQR) for each RIGHT domain were as follows: basic information (4, 1), background (3, 2), evidence (0, 0), recommendations (2, 2), review and quality assurance (0, 0), funding and declaration and management of interests (0, 0.5), and other information (0, 0). (2) The items with a low reporting rate (<10%) included 2, 5, 8b, 9a, 10a, 10b, 11a, 11b, 14a, 14b, 14c, 16, 17, 19b, 20, 21, and 22, and those with a high reporting rate (> 90%) included 1a, 1b, 1c, 7b, 13a, and 13b. (3) In recent years, the reporting quality of TCM CPGs has improved, and there was a significant difference among the organizations (P = 0.000), where that of the updated versions was greater than that of the historical versions (P = 0.047). (4) The RIGHT and AGREE II scores were positively correlated (P = 0.014).

**Conclusions:**

At present, although the reporting quality of TCM CPGs is improving, the overall quality remains suboptimal. Guideline developers should strictly follow the evidence-based process of developing guidelines and should follow the RIGHT statement to produce a standardized report when writing guidelines.

## Introduction

Clinical practice guidelines (CPGs) are statements that include recommendations that are intended to optimize patient care and are informed by a systematic review of evidence and an assessment of the benefits and harms of alternative care options[1]. Guidelines are an important basis on which medical workers make clinical decisions, and CPGs are the criteria for standardizing diagnosis and treatment. With the development of evidence-based medicine, the number of guidelines is increasing, and the reporting standards are very important. On the one hand, the standardized reporting of guidelines can improve the science and transparency and can help control the risk of bias in the guideline development process. On the other hand, standardized reporting is crucial for promoting the reading and utilization of the guidelines. Standardized guidelines will help users more quickly and accurately grasp the specific content and make comprehensive and objective judgments based on the guidelines.

In November 2016, “A Reporting Tool for Practice Guidelines in Health Care: The RIGHT Statement”(checklist is presented in S1 Appendix)[2], was published in the Annals of Internal. The international multidisciplinary team of RIGHT (Reporting Items for practice Guidelines in Healthcare) joined more than 20 experts from 11 countries, including China, the United States, Canada, the United Kingdom and Germany, and from seven international organizations, including the WHO (World Health Organization), EQUATOR (Enhancing the QUAlity and Transparency Of health Research), GIN (Guidelines International Network), The Cochrane Library, GRADE (The Grading of Recommendations Assessment, Development and Evaluation) and AGREE. This research lasted 3 years to develop in strict accordance with the international health research reporting standards, based on the WHO guidelines, an analysis and summary of the COGS (Conference on Guideline Standardization) and AGREE II items. The RIGHT statement contains 7 major domains and 22 items. It is the first international reporting standard to be formally registered on the EQUATOR network and is currently the only reporting standard applicable to CPGs, public health guidelines and health policy guidelines.

Traditional Chinese medicine (TCM), as a representative of traditional medicine, plays an indispensable role in the Chinese healthcare system[3] and has a widespread impact on the world[4]. At present, it is of great significance to evaluate the reporting quality of TCM CPGs. In this study, we utilized the RIGHT statement to evaluate the currently published TCM CPGs. We also aimed to determine ways to improve the reporting quality and discuss the applicability of RIGHT to TCM guidelines.

## Methods

We used systematic review methods in this study.

### Search strategy

We searched the following databases: SinoMed, China National Knowledge Infrastructure (CNKI), Wanfang and PubMed, and the guideline clearinghouses of National Guideline Clearinghouse (NGC), Guidelines International Network (GIN), the National Institute for Health and Care Excellence (NICE), and Medlive. We searched Google, Amazon, and Dangdang to obtain guidelines published in books, and we searched the supplemental references. The deadline was July 2017, and the language restrictions were Chinese or English. A search strategy using the keywords “consensus”, “statement”, “recommendation”, “guideline”, “Chinese medicine”, “Chinese herbal”, and “TCM” was employed (full search strategies are presented in S2 Appendix).

### Inclusion and exclusion criteria

The inclusion criteria were reports on Chinese medicinal compounds or recommendations for proprietary Chinese medicine interventions. The exclusion criteria were as follows: (1) integrative medicine, acupuncture and moxibustion guidelines; (2) other supplementary alternative medicine guidelines; (3) modern medicine guidelines; and (4) repeating guidelines.

### Evaluation of reporting quality

Eight trained researchers (TYJ, WZJ, XYJ, CG, DYL, HJH, ZQ, XY) formed four groups, each of which evaluated part of the guidelines. The researchers compiled a data extraction form according to the RIGHT statement, extracted data independently in Excel and analyzed the reporting quality according to 22 items (35 subitems in total). We combined the RIGHT items in accordance with a research paper by one of the RIGHT statement authors[5]. After a group discussion, we decided to make a judgment of “reported,” “partially reported” and “unreported,” with corresponding scores of 1 point, 0.5 point or 0 points. All evaluation processes were conducted independently, and if there was a disagreement, it was resolved by reaching a consensus. Finally, we summarized the results, analyzed the reporting scores or rates in each domain and item and calculated the reporting quality by subgroup analysis.

### Statistical analysis

We conducted a descriptive statistical analysis to calculate the score and reporting rate. The Mann-Whitney test was used to compare two samples, and the Kruskal-Wallis test was used to compare multiple samples; a value of P<0.05 denoted statistical significance. The intraclass correlation coefficient (ICC) was used to test the interrater reliability among the reviewers.

### Quality control

Before the evaluation, eight researchers studied the RIGHT statement together to ensure that each researcher had a consistent understanding. We conducted two pretests (12 TCM guidelines randomly selected by computer) and calculated the ICC using SPSS 19.0. The results were as follows: ICC1 = 0.962, 95% CI: [0.953, 0.969]; ICC2 = 0.973, 95% CI: [0.967, 0.978].

## Results

**Fig 1:**
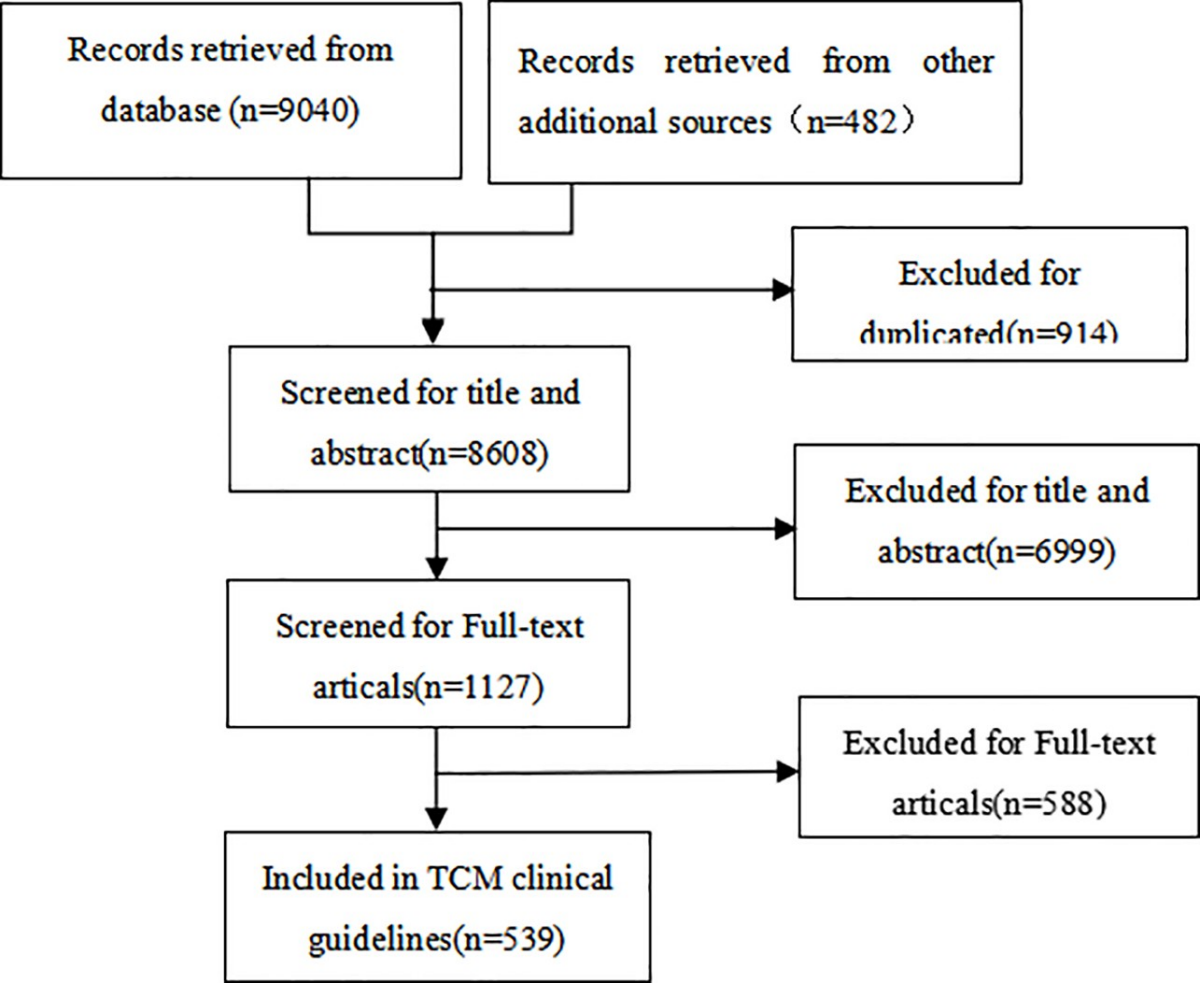
Flow diagram of the selection process for the studies.

Overall, 539 TCM CPGs were included. A diagram of the screening process is shown in Fig 1.

### Guideline characteristics

The 539 TCM CPGs covered a range of topics. The guideline characteristics are shown in Table 1.

**Table 1:** Characteristics of 539 TCM CPGs.

Categories	n (%)
**Publication**	
Journal	57 (10.58%)
Book	482 (89.42%)
**Type of disease**	
Modern medical disease	344 (63.82%)
TCM disease	193 (35.81%)
Modern medical disease also TCM disease	1 (0.19%)
TCM syndromes	1 (0.19%)
**Organization**	
China Association of Chinese Medicine (CACM)	457 (84.79%)
World Health Organization’s Western Pacific Organization (WHO WPRO)	28 (5.19%)
CACM chapter	25 (4.63%)
Hospitals	17 (3.15%)
Government	4 (0.74%)
Universities	2 (0.37%)
Other societies	6 (1.11%)
**Type of version**	
Historical	8 (1.48%)
Updated	8 (1.48%)

### Domain scores

**Fig 2:**
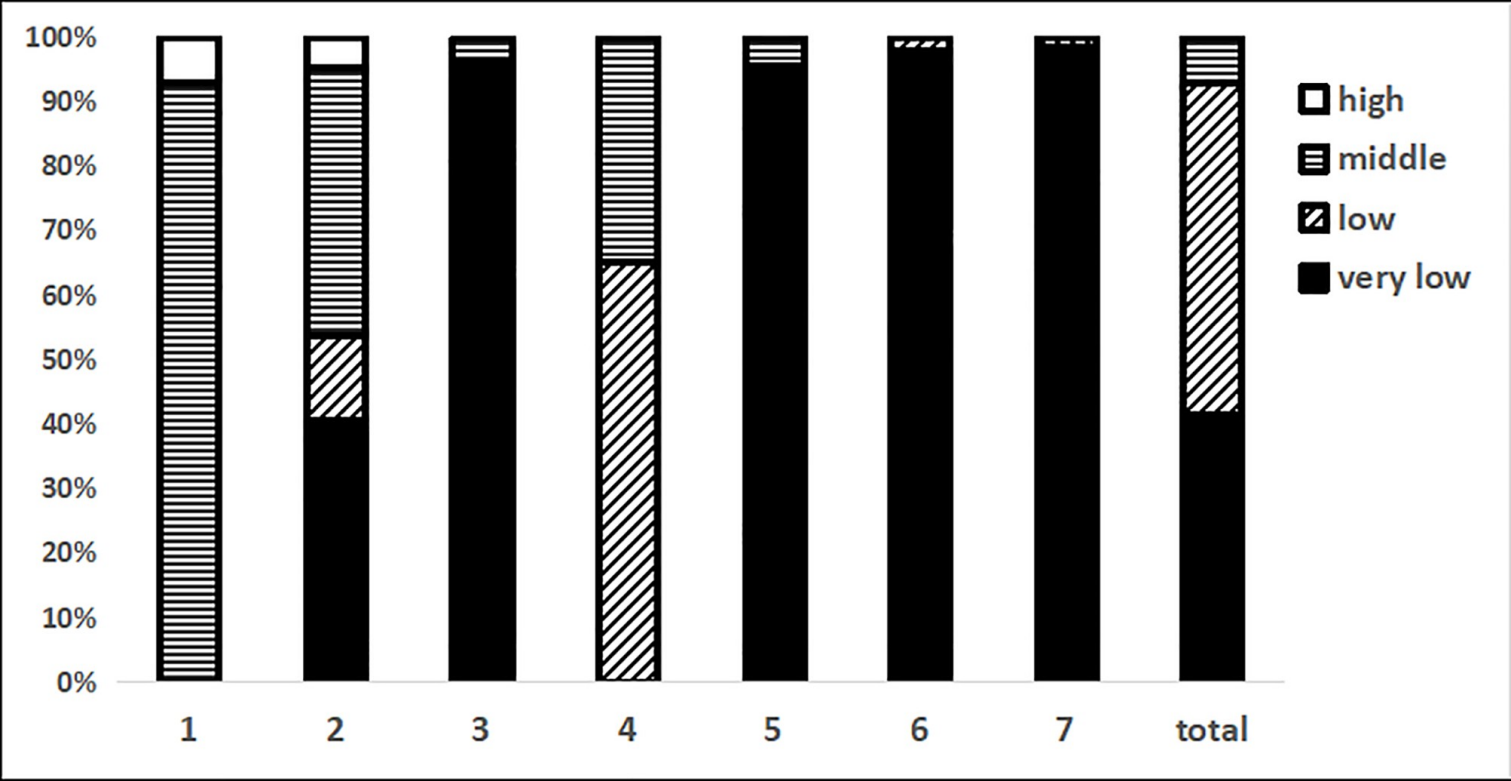
The distribution of domain scores.

The score distributions of the domains are shown in Fig 2. We divided the scores into four levels: high (≥75%), middle (50–75%), low (25–50%), and very low (≤25%). The results showed that (1) the domain 1 (basic information) scores were higher than the other scores and were mainly distributed in the middle levels. (2) The domain 2 (background) scores were mainly distributed in the very low and middle levels. (3) In domain 4 (recommendations), the scores were all in the range of the low and middle levels. (4) The scores of domains 3 (evidence), 5 (review and quality assurance), 6 (funding and declaration and management of interests), and 7 (other information) were distributed in the very low level range. (5) Overall, 92.9% of the guidelines were distributed between the very low and low levels, with only 7.1% having middle level scores; no guidelines had a score at the high levels.

### The reporting rate of items

**Fig 3:**
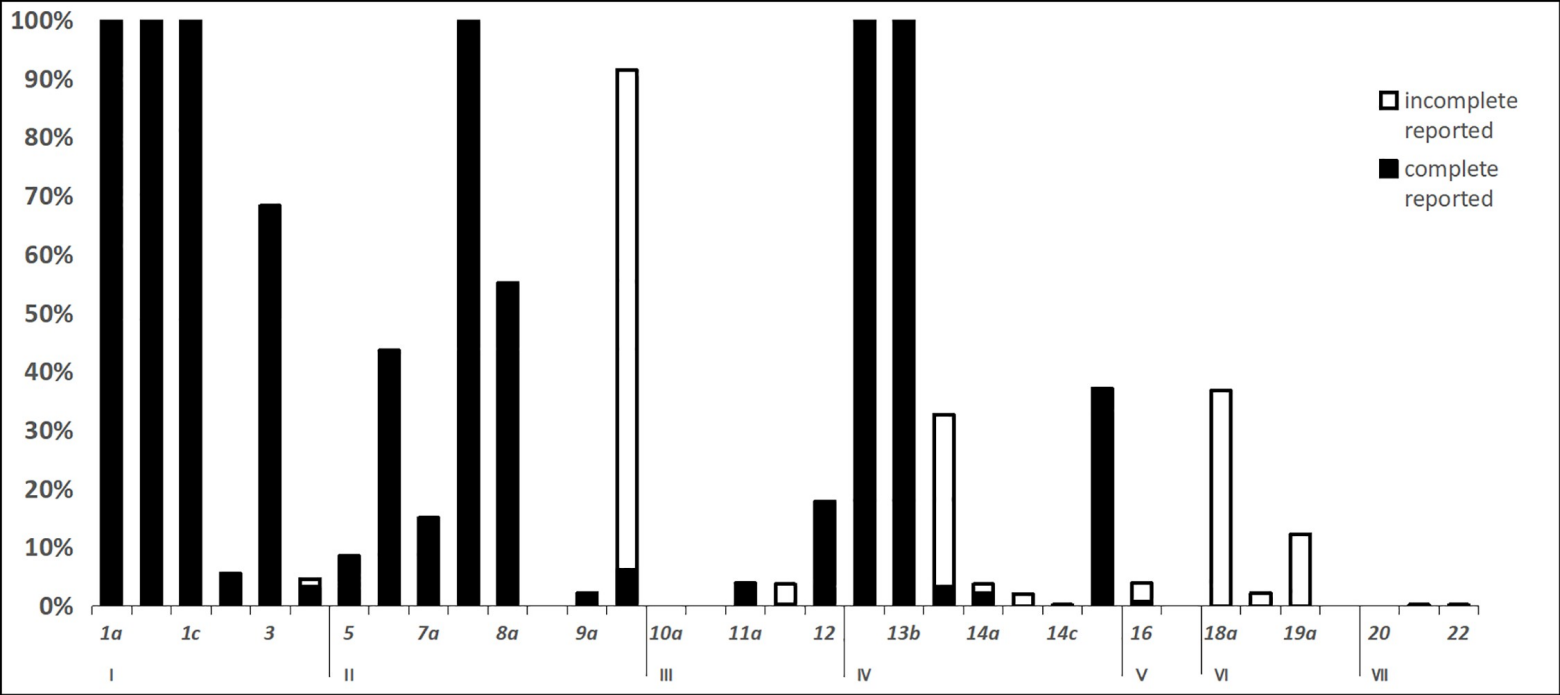
The reporting rates of items.

The reporting rates of the items are shown in Fig 3. The results showed that the items with a low reporting rate (<10%) were 2, 5, 8b, 9a, 10a, 10b, 11a, 11b, 14a, 14b, 14c, 16, 17, 19b, 20, 21, and 22 and that the high-reporting-rate (> 90%) items were 1a, 1b, 1c, 7b, 13a, and 13b. For the low to high reporting rates, (1) the reporting rates of items 8b, 10a, 10b, 17, 19b, and 20 were all 0%, indicating that there was no guideline to report the above items; these items were mainly distributed among the implementation, PICO questions, conflict of interest, quality assurance and accessibility of the guidelines. (2) The reporting rates of items 14c, 21 and 22 were 0.19%, indicating that only 1 guideline reported the items; these items were mainly distributed among the fairness and feasibility of the recommendations, acceptability, limitations and research gaps. (3) The reporting rates for items 2, 5, 9a, 11a, 11b, 14a, 14b and 16 were also below 10%, and these items mainly concerned the executive summary, epidemiology, group members, evidence, patient preferences and values, cost-effectiveness, and external review. (4) The reporting rates of 9b, 13c, 18a, and 19a were > 10%, but the full rates were all less than 5%, and these items were mainly distributed among the strength of recommendation, quality of evidence, and funding. For example, 9b had a reporting rate of 97.59%; thus, most of the guidelines reported the personal information of members, but job, work and other information were not completely reported. (5) For items 3, 8a, and 15, the reporting rates were 68.27%, 55.10%, and 37.11%, respectively, and these items concerned abbreviations, purpose, the decision-making process and methodology. (6) The best reporting rate was observed for items 1a, 1b, 1c, 7b, 13a, and 13b, at 100%, and these items were mainly distributed among basic information, the subgroups and providing precise and actionable recommendations.

### Subgroup analysis

According to the different stratified criteria (including the year of publication, organization, and type of version used to evaluate the reporting quality), the results (Table 2) showed the following: (1) according to the year of journal publication, the reporting quality score of each year was divided into three levels: 2003–2009, 2010–2015, and 2016–2017. The reporting quality of the guidelines increased over time. However, domains 1 and 7 did not increase, and these were the basic information and other information domains. In addition, we found that the guidelines published in 2016–2017 were all based on evidence-based methods. (2) There was a significant difference in the reporting quality among the organizations (P<0.001). Among them, the scores for the WHO, WPRO and hospitals were higher than those for other organizations. (3) Regarding the type of version, only 8 guidelines were updates of historical guidelines. The Mann-Whitney test (P = 0.047) showed that the reporting quality of an updated version was higher than that of the corresponding historical version. The scores with the greatest increases were in domains 2, 4, 5, and 6, which were member selection, recommendations, review and dissemination. The scores of domains 1, 3, and 7 did not increase, and these domains concerned basic information, evidence, accessibility, limitations and research gaps.

**Table 2:** RIGHT domain scores of different subgroups.

Subgroups	The domain scores (Med, IQR)							
	Basic information	Background	Evidence	Recommendations	Review and quality assurance	Funding and declaration and management of interests	Other information	Total scores
**Organization**								
CACM(N = 457)	4.00, 1.00	3.00, 2.00	0.00, 0.00	2.00, 2.00	0.00, 0.00	0.00, 0.50	0.00, 0.00	9.00, 5.50
WHO WPRO(N = 28)	5.50, 0.38	6.50, 0.75	1.75, 1.50	4.50, 0.38	0.00, 1.00	0.50, 0.50	0.00, 1.00	19.50, 1.00
CACM Chapter(N = 25)	4.00, 0.00	4.50, 2.00	0.00, 1.00	2.00, 1.00	0.00, 0.00	0.00, 0.50	0.00, 0.00	11.50, 5.00
Hospital(N = 17)	4.00, 0.50	5.50, 0.50	1.00, 0.75	5.00, 1.50	0.00, 0.50	1.50, 1.00	0.00, 0.00	18.00, 4.00
Others(N = 6)	4.00, 1.63	3.75, 1.50	0.00, 0.25	3.00, 0.50	0.00, 0.00	0.00, 1.00	0.00, 0.00	11.50, 4.25
University(N = 2)	4.00, 0.00	4.00, 0.00	1.25, 0.00	3.00, 0.00	0.00, 0.00	0.75, 0.00	0.00, 0.00	13.00, 0.00
Government(N = 4)	3.00, 0.00	2.00, 1.88	0.00, 0.00	2.00, 0.75	0.00, 0.00	0.00, 0.00	0.00, 0.00	7.50, 2.13
P-value	<0.001	<0.001	<0.001	<0.001	<0.001	<0.001	<0.001	<0.001
**Version**								
Historical(N = 8)	4.00, 0.75	4.00, 1.50	1.00, 1.00	4.00, 0.75	0.00, 0.00	1.00, 0.88	0.00, 0.00	14.00, 4.88
Updated(N = 8)	4.00, 0.00	5.50, 1.50	1.00, 1.50	5.00, 0.75	1.00, 0.75	1.50, 0.75	0.00, 0.00	18.00, 6.75
P-value	0.440	0.007	0.069	0.015	0.003	0.021	1.000	0.047
**Year (in journal)**								
2003-2009(N = 10)	4.00, 0.25	2.75, 1.63	0.00, 0.00	2.00, 0.00	0.00, 0.00	0.00, 0.13	0.00, 0.00	8.75, 2.00
2010-2015(N = 31)	4.00, 1.00	4.50, 2.00	0.00, 1.00	3.00, 1.00	0.00, 0.00	0.50, 0.50	0.00, 0.00	12.50, 4.50
2016-2017(N = 16)	4.00, 0.38	5.50, 0.75	1.00, 1.13	5.00, 1.75	1.00, 1.00	1.50, 1.38	0.00, 0.00	18.00, 4.25
P-value	0.852	<0.001	<0.001	<0.001	<0.001	<0.001	0.657	<0.001

### Comparison with the AGREE II score

**Fig 4:**
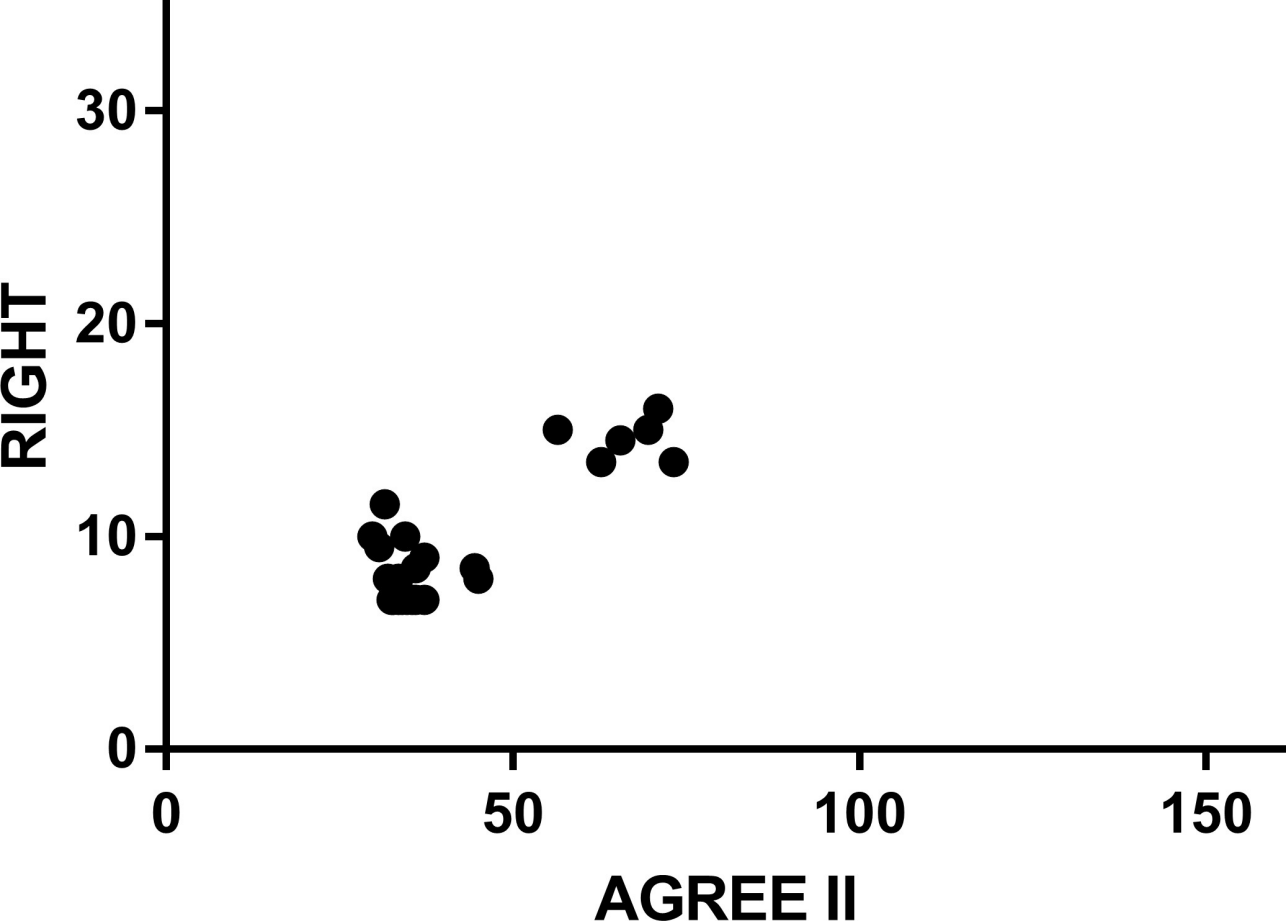
Scatter plot for the correlation between RIGHT and AGREE II scores.

AGREE II, a methodology assessment tool for guidelines, was published by AGREE Research Teams and focuses on the methodological quality of guidelines in the development process. RIGHT, a reporting quality assessment tool for guidelines, was published by the RIGHT Group and focuses on the reporting quality of the guideline’s content. L Yao et al.[6] used AGREE II to evaluate the CPGs of TCM. To determine the relationship between the reporting quality and the methodological quality, we obtained the original data from the authors and analyzed the correlation between the AGREE II scores and the RIGHT scores of 43 TCM guidelines. The total score obtained with RIGHT was 35. The total score obtained with AGREE II was 161. The Spearman correlation analysis using the SPSS software showed that R = 0.371, 95% CI [0.070, 0.610], P = 0.014 (the scattergram is shown in Fig 4), and the results showed that there was a positive correlation between the AGREE II and RIGHT scores.

## Discussion

Over the past 25 years, with the development of evidence-based medicine, guidelines have become a powerful tool for establishing the best dataset of health evidence[7]. The number of TCM CPGs has also increased[8], and studies have shown that TCM CPGs still have some problems with quality and applicability[9]. We collected as much literature as we could, and from the results, we found the following problems. First, for the guideline type, TCM CPGs pay more attention to the diagnosis and treatment of diseases and mention other aspects, such as disease prevention or patient version guidelines, less frequently. Second, regarding different organizations, there is a certain reporting quality gap between the guidelines published by China and those published by international organizations. Third, Low-quality reported items have the following aspects: (1) These items lack a summary of the recommendations, which can help users grasp the main contents of the guideline quickly and accurately, and this may be related to the limited length of the report in journals; thus, the accessibility of such guidelines is important. (2) There is a lack of epidemiology, especially for TCM diseases. (3) There is incomplete reporting of the information on group members, including their personal information, work, contribution and selection method. (4) There is a lack of description of the PICO question, a lack of search strategies, screening criteria and quality assessment, and less consideration of patient preferences and values and cost-effectiveness. (5) There is a lack in reporting conflicts of interest, including a detailed description of the conflict type, the stage, the funder's role, assessment and accessibility[10]. (6) There is a lack of reporting on dissemination, implementation, updating, accessibility and limitations.

The ultimate goal of the guidelines is to guide clinical practice. A study has shown that low-quality guidelines may cause harm to patients, waste medical resources and affect medical decisions[11]. Through a reporting tool, we can present the development process more comprehensively and accurately. We analyzed that the current low quality of TCM CPGs may be due to the following reasons. (1) The lack of a developed method. Currently, many organizations have released the method that they used to develop guidelines, such as NICE and the WHO. Guideline developers may not yet have adopted international methods, with most guidelines based only on expert consensus[12]. (2) Unclear PICO questions. This may be related to the specificity of TCM; therefore, how to combine the PICO model with the TCM guidelines is a question worth exploring. (3) A lack of evidence based on systematic reviews. In 2011, the Institute of Medicine (IOM) clearly defined “CPGs as statements that include recommendations that are intended to optimize patient care and are informed by a systematic review of evidence and an assessment of the benefits and harm of alternative care options”[1]. Only 3.90% of the guidelines reported the use of systematic reviews as a source of evidence in the development process, which may be related to the lack of high-quality research. (4) A lack of evidence quality and recommendation strength. Medical practice should be based on the best available evidence and should make a clear and strong recommendation by considering different benefits and harm, patient preferences and values, cost-effectiveness, feasibility, acceptability, and fairness[13]. Most of the guidelines did not report these aspects because there is currently no widespread classification system for evidence quality and recommendations in TCM. (5) It is often difficult to obtain the full text, appendix and related documents concerning the guidelines.

To improve the reporting quality of TCM CPGs, we propose the following suggestions. (1)Strengthen the dissemination of the RIGHT statement, introduce the RIGHT statement into medical journals, and standardize the writing of guidelines. (2) Strengthen the methodological training of developers, recommend that guideline developers use evidence-based methods. change the members from clinicians to multidisciplinary teams including methodologists. (3) Pay more attention to the patient’s values and preferences, consider the cost-effectiveness of the development process to benefit and reflect the TCM specialty, strengthen the management of conflicts of interest. (4)Improve the implementation of guidelines. (5) Establish a TCM guideline clearinghouse to improve the accessibility of the guidelines. (6) Regularly update the guidelines and report the update mechanism.

## Conclusion

Through the analysis of 539 TCM clinical guidelines, our study showed that the reporting quality for various domains and items is different. Although the reporting quality of TCM CPGs is improving, the overall quality remains suboptimal. There is a close relationship between the reporting quality and the methodological quality of the guidelines. Guideline developers should follow the RIGHT statement to produce a standardized report when writing guidelines.

## Limitations

There are some limitations to our study. Only the TCM guidelines published in books and journals were searched, so guidelines published elsewhere may have been omitted. This study only aimed to evaluate TCM guidelines; integrative medicine, acupuncture and other traditional therapies were not included. For the guidelines published in journals, due to space constraints, it may have been difficult to show all the methodological information about the process.

## Suggestions for future research

TCM is one of the oldest medical systems in the world. In TCM, the understanding of disease differs from that used in modern medicine. TCM has the unique treatment principles of “treatment based on syndrome differentiation” and the “concept of holism”[14]. TCM guidelines also have their own distinctive features and complex patterns; in the case of diseases that have been classified, there is also a wide variety of situations, such as using Chinese medicine for modern diseases, using Chinese medicine for TCM diseases, and using Chinese medicine for TCM syndromes. We also had some difficulty in using the RIGHT tool, as TCM has its own unique historical background. In addition, regarding epidemiology, statistics are difficult to obtain for TCM diseases. The recommendations lack content concerning theory, principles, formulas, and medicines[15]. In terms of evidence, there is no uniform reporting standard for grading the evidence quality of ancient literature. Because of these aspects, the existing RIGHT statement has not been widely applied. A TCM CPG should be more effectively combined with foreign methods while maintaining its own characteristics, and a reporting standard applicable to TCM guidelines should be established and is worth further exploration.

## Supporting information

S1 File Original data-information of guidelines,score result. (SAV)

S2 File PRISMA 2009 checklist. (DOC)

S1 Appendix (DOCX)

S2 Appendix (DOCX)
