# Supplementary material for: Using the RIGHT statement to evaluate the reporting quality of clinical practice guidelines in traditional Chinese medicine
Source: PLoS One. 2018 Nov 16;13(11):e0207580. doi: 10.1371/journal.pone.0207580 (PMC6239316; doi:10.1371/journal.pone.0207580)
Supplement: S2 Appendix — (DOCX) [file pone.0207580.s004.docx]

**Appendix 1. Search strategies for TCM guidelines**

1. **PubMed（1966-2017.7）**

#1 "Medicine, Chinese Traditional"[Mesh]

#2 “Chinese medicine” [Title/Abstract]

#3 “Chinese herbal” [Title/Abstract]

#4 “TCM”[Title/Abstract]

#5 OR/#1-4

#6 “Guidelines as topic” [Mesh]

#7 “guideline” [Title/Abstract]

#8 “recommendation” [Title/Abstract]

#9 “consensus” [Title/Abstract]

#10 “statement” [Title/Abstract]

#11 "Guideline" [Publication Type]

#12 OR/#6-11

#13 #5 and #12

1. **Wanfang Data Knowledge Service Platform （1988-2017.7）**

#1 指南[主题]

#2 共识[主题]

#3 OR/#1-2

#4 中医[主题]

#5 中药[主题]

#6 传统医学[主题]

#7 OR/#5-7

#8 #3 and #7

1. **China National Knowledge Infrastructure（1999-2017.7）**

#1 指南[主题]

#2 共识[主题]

#3 OR/#1-2

#4 中医[主题]

#5 中药[主题]

#6 传统医学[主题]

#7 OR/#5-7

#8 #3 and #7

1. **SinoMed（1978-2017.7）**

#1 "指南"[不加权:扩展]

#2 "指南"[常用字段:智能]

#3 "共识"[常用字段:智能]

#4 OR/#1-3

#5 "中医药"[不加权:扩展]

#6 "医学，中国传统"[不加权:扩展]

#7 "中医"[常用字段:智能]

#8 "中药"[常用字段:智能]

#9 "传统医学"[常用字段:智能]

#10 OR/#5-9

#11 #4 and #10

1. **Medlive（2006-2017.7）**

#1 中医药

1. **NGC (national guideline clearinghouse) （1998-2017.7）**

#1 Traditional Chinese medicine

1. **GIN (Guidelines International Network)（2002-2017.7）**

#1 Traditional Chinese medicine

1. **NICE (National Institute for Health and Care Excellence)（1999-2017.7）**

#1 Traditional Chinese medicine

1. **Google**

“Traditional Chinese medicine” and Guidelines (the first 200 records were screened)

1. **Amazon**

“Guidelines” were searched as key word for TCM guidelines published as books

1. **Dangdang**

“Guidelines” were searched as key word for TCM guidelines published as books
